# Analytical Method Cluster Development for Comprehensive Characterisation of Suberinic Acids Derived from Birch Outer Bark

**DOI:** 10.3390/molecules28052227

**Published:** 2023-02-27

**Authors:** Daniela Godina, Raimonds Makars, Aigars Paze, Janis Rizhikovs

**Affiliations:** 1 Biorefinery Laboratory, Latvian State Institute of Wood Chemistry, LV-1006 Riga, Latvia; 2 PolyLabs SIA, Mukusalas iela 46, LV-1004 Riga, Latvia

**Keywords:** suberin, suberinic acids, biomass, birch bark, depolymerisation, biorefinery

## Abstract

Suberin is a complex polyester biopolymer, and it is practically impossible to estimate the real content of suberin in suberised plant tissues. This indicates the importance of the development of instrumental analytical methods for the comprehensive characterisation of suberin derived from plant biomass for the successful integration of suberinic products into biorefinery production chains. In this study, we optimised two GC-MS methods—one with direct sylilation, and the second with additional depolymerisation, using GPC methods with RI detector and polystyrene calibration and with a three-angle light scattering detector and an eighteen-angle light scattering detector. We also performed MALDI-Tof analysis for non-degraded suberin structure determination. We characterised suberinic acid (SA) samples obtained from birch outer bark after alkaline depolymerisation. The samples were particularly rich in diols, fatty acids and their esters, hydroxyacids and their corresponding esters, diacids and their corresponding esters, as well as extracts (mainly betulin and lupeol) and carbohydrates. To remove phenolic-type admixtures, treatment with ferric chloride (FeCl_3_) was used. The SA treatment with FeCl_3_ allows the possibility to obtain a sample that has a lower content of phenolic-type compounds and a lower molecular weight than an untreated sample. It was possible to identify the main free monomeric units of SA samples by GC-MS system using direct silylation. By performing an additional depolymerisation step before silylation, it was possible to characterise the complete potential monomeric unit composition in the suberin sample. For the molar mass distribution determination, it is important to perform GPC analysis. Even though chromatographic results can be obtained using a three- laser MALS detector, they are not fully correct because of the fluorescence of the SA samples. Therefore an 18-angle MALS detector with filters was more suitable for SA analysis. MALDI-Tof analysis is a great tool for the polymeric compound structural identification, which cannot be done using GC-MS. Using the MALDI data, we discovered that the main monomeric units that makes up the SA macromolecular structure are octadecanedioic acid and 2-(1,3-dihydroxyprop-2-oxy)decanedioic acid. This corresponds with GC-MS results, showing that after depolymerisation hydroxyacids and diacids were the dominant type of compounds found in the sample.

## 1. Introduction

The European Union (EU) recognises that bio-based materials are critical to the creation of a more circular and decarbonised economy as well as the transition from a fossil to a bio-based economy [1]. The development of advanced new materials and technologies for bio-based products is critical as the world faces a growing number of issues, leading to growing public awareness about global sustainability [1,2,3].

The outer bark of most birch species, widespread in northern Europe, is rich in pentacyclic lupane-type triterpenes, mainly betulin and lupeol, which can be extracted in good yields from birch outer bark [4,5]. The birch outer bark contains up to 50% (dry basis) of suberin [6,7]. Therefore, after triterpene extraction, the remaining biomass can still be used for the production of other high-added-value products.

**Figure 1:**
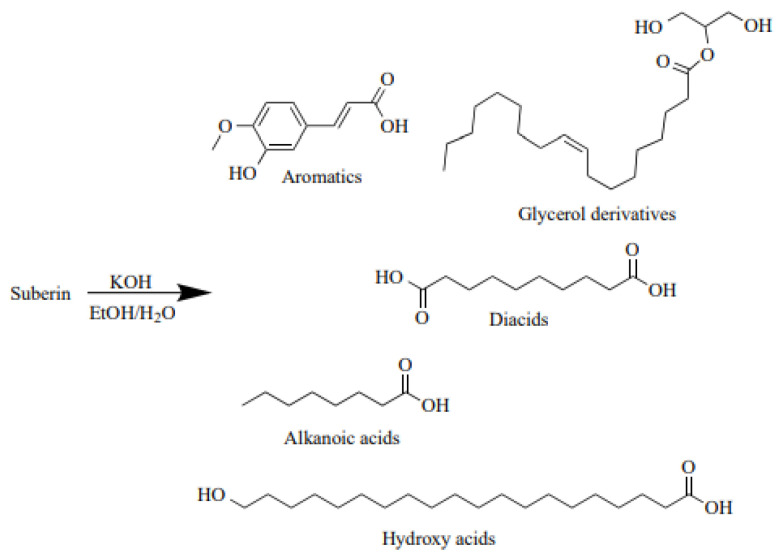
Possible suberin depolymerisation products in alkaline media [16].

Suberin is a lipophilic macromolecule that shields plants from pests and the environment. It is found in the cell walls of many plants’ outer layer (bark) [8,9,10,11]. It is a complex polyester made of glycerol, covalently bonded to lignin monomer-like polyphenols in the birch outer bark, and linear, polyfunctional long carbon chain fatty acids-suberinic acids (SA). Various SAs are produced when suberin is depolymerised in alkaline media (Figure 1). SAs are long, bifunctional saturated, and unsaturated fatty acids that are a subset of carboxylic acids that have a strong adhesion to wood after being heated [12]. Omega-hydroxyfatty acids, alpha, and omega-dicarboxylic acids, as well as analogous mid-chain di-hydroxy or epoxy derivatives, make up most suberin’s aliphatic domains, while differently substituted phenolic moieties dominate the aromatic domains [13,14]. Because of the complicated macromolecular structure of suberin and the structural resemblance between its aromatic domains and lignin, it is almost impossible to measure the true quantity of suberin in suberised plant tissues [15].

A combination of SAs can be used to produce wood-based panels. A natural and risk-free substance for human health is a binder made of SA. The finished products feature excellent bending strength, internal bond strength, and dimensional stability in addition to being moisture-resistant [17,18]. Some information on the use of the suberin depolymerisation products as monomers for the synthesis of novel macromolecular materials have been reported. Among such macromolecular materials are polyurethanes synthesised using the mixture of aliphatic monomers resulting from the methanolysis process used to cleave the suberin ester moieties in their methyl ester form. In our previous publication we also tested the use of SA for bio-polyol synthesis [16] and concluded that SAs have a high potential in bio-polyol production because they have high hydroxyl values typical and necessary for polyols to be used for rigid PU foam production. However, the apparent viscosity of bio-polyols was too high. Therefore, to be able to use these polyols in the production of rigid PU foams, further studies are being conducted. Suberin samples also contain polyphenols, which cause the dark colour of a depolymerised SA mixture and are responsible for self-polymerisation, which is undesirable for further processing as a raw material in polymer synthesis and in coatings or impregnation processes [16]. In our previous work using the Folin-Ciocalteu method, we determined that SA samples derived from birch outer bark contain up to 5% of polyphenolic compounds [16]. Therefore, the possibility of purification SA with ferric chloride (FeCl_3_) was investigated in this research by precipitating the tannins from depolymerised SA before acidification. N. Kumara Swamy et al. [19] discussed a possible mechanism for the removal of phenols using FeCl_3_. Adsorption of phenolate ions in aqueous media by metal ions to create metal phenolates is the mechanism by which phenolic derivatives are chemically precipitated. When the pH is very high, the rate of phenol precipitation increases. Phenols becoming more ionised into phenolate ions under strongly alkaline conditions might cause this. The larger availability of phenolate ions at higher pH levels increases the conversion of metal ions into metal phenolates [20].

SAs can be characterised using different instrumental methods, most typical being size exclusion chromatography (GPC) for molar mass distribution determination, gas chromatography (GC) for monomeric compound identification, and Fourier-transform infrared spectroscopy (FTIR) for general functional group determination in SA samples. The identification of suberin monomers by GC equipped with a mass spectrometry detector, usually after derivatisation to the corresponding TMS (trimethylsilyl) esters, in most research has been limited to qualitative analysis and not quantification, mostly because of the limitations of standard substance availability. GC analysis provides information only about the monomeric fraction of the suberin, and that is why it is important to use different chromatography methods, such as GC and GPC to obtain information about polymeric fractions.

In summary the main purpose of this work was to develop an analytical method cluster for comprehensive characterisation of SA samples derived from plant biomass. The approach was essential for further successful integration of suberinic products into biorefinery production chains, and to compare the composition of the purified and unpurified SA to predict their further potential in obtaining polymeric materials.

## 2. Results and Discussion

### 2.1. SA Sample Characteristics

To chemically characterise SA sample acid number, epoxy group and total phenolic content (TPC) were determined (Table 1). It can be seen that for the treated sample (SA_T) acid number and epoxy group content was higher than these values in the untreated sample (SA_U). This can be explained by the isolation of reactive phenolic (TPC content decreased) compounds that did not participate in the self-polymerisation process of SA after drying at elevated temperature (80 °C). The decrease in TPC indicates that in the sample preparation step some amounts of phenolic compounds (polyphenolic) were removed after the treatment with FeCl_3_. Consequently, the removal of phenolic compounds from the SA resulted in lower yield.

**Figure 2:**
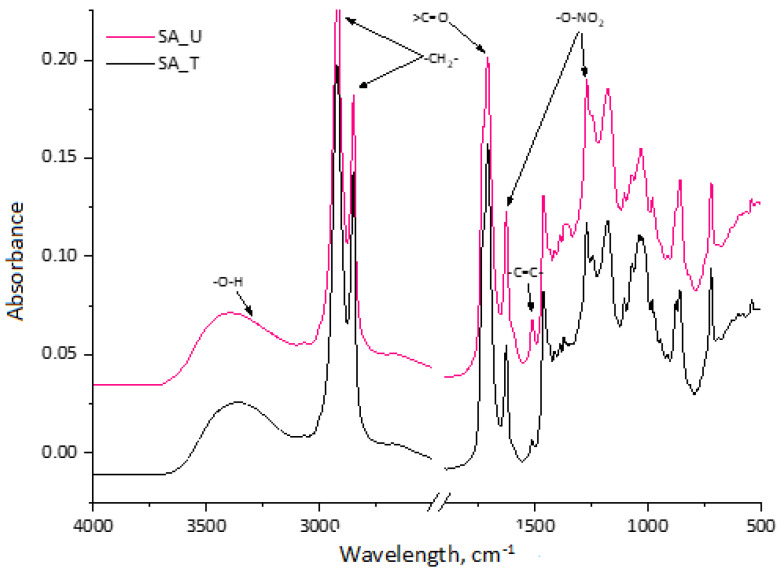
FTIR spectra of treated and untreated SA samples.

Treated and untreated SA samples were analysed with FTIR (Figure 2). The broad absorption peak between 3600 and 3100 cm^−1^ of spectra of the obtained SA fractions was identified as characteristic stretching vibrations of the –O–H group [16]. Symmetric and asymmetric –CH_2_– stretching peaks were observed at ~2930 and 2855 cm^−1^, which point to the long aliphatic chains of suberin and SA [21].

The strong peak at 1710 cm^−1^ represents carboxylic acid groups, which were similar in both samples. A small shoulder of this peak also appeared at ~1730 cm^−1^, indicating that some part of SA bonded with glycerol in esters type compounds. The peak at 1510 cm^−1^ indicates a C=C stretch from the conjugated carbonyl groups, which are typically aromatic. They are attributed to polyphenolic compounds from both tannins and lignin degradation products. A more pronounced peak for the untreated sample (SA_U) was observed corresponding to the higher TPC in Table 1. The peaks at 1463 cm^−1^ and 1375 cm^−1^ indicate asymmetric and symmetric C–H deformations of the aliphatic regions, which are characteristic of suberin-derived aliphatic acids. The symmetric and asymmetric stretching vibrations of –O–NO_2_ at 1270 cm^−1^ and 1625 cm^−1^ could be seen in the spectra. This indicates that the SA sample contained a noticeable residue of nitrate salt leftovers from the bark depolymerisation process.

### 2.2. Suberin Monomers from Birch Bark

For suberin monomeric compound determination we developed a GC-MS method. In most of the reported studies [16,22] of suberin composition, the quantification of individual suberin monomers was done using the peak areas of the MS or FID signals. However, differences in the response factors and electron-impact fragmentation of the analysed monomers can introduce significant inaccuracies in the quantification data. Therefore, we used standard calibrations for the monomer quantification. As described in Section 3.6., we used several standard substances covering different groups of compounds (diols (1), fatty acids (2), hydroxyacids and their esters (3), diacids and their esters (4), extractives (5), aromatics (6) and carbohydrates (7)). For each calibration graph, the corresponding equation is given in Table 2. Calculated GC method parameters (linearity range, R^2^, LOD and LOQ) are also shown in Table 2. As seen from the calculations, the GC-MS method can be used to reliably quantify SA chemical composition, even in small concentrations.

Direct sample silylation was used to determine the suberin monomeric constituents (Method 1).

**Figure 3:**
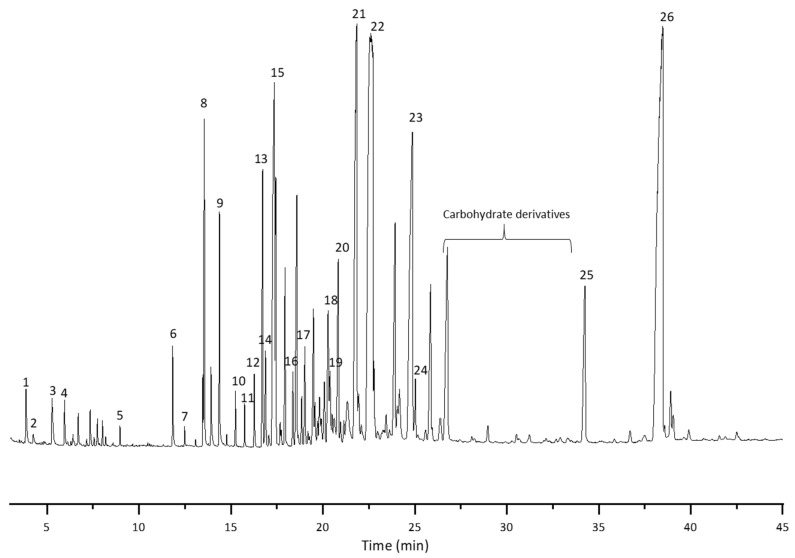
Total ion chromatogram of the SA sample (SA_U). The peak number refers to the compounds listed in Table 3.

Figure 3 depicts the gas chromatogram of the TMS ester derivatives of sample SA_U. When comparing to the chromatogram obtained from SA_T sample (not shown), similar qualitative composition but different quantitative ratios of suberin monomers were observed. Table 3 shows the identified suberin monomers and the molecular weight of the corresponding silyl derivatives. Additionally, the identified compounds were attributed to the corresponding group.

In both suberin samples, different fatty acids, hydroxyacids, diacids and their corresponding esters were identified. Some pentacyclic triterpenoids, such as lupeol and betulin, along with smaller amounts of other unidentified triterpenoids were detected at longer retention times. Even if the extraction process was carried out for the birch outer bark before the depolymerisation step, up to 10% triterpenoids were still present in the feedstock’s intercellular space. After the depolymerisation, the birch outer bark cell structure is degraded and thus triterpenoid extractives can be found in the isolated SA samples. Also some aromatics and carbohydrates can be found in the samples.

After the Method 1 the main suberin components identified by GC-MS were fatty acids (linoelaidic acid ethyl ester and ethyl stearate), hydroxyl acids (2-hydroxy-decenedioic acid and 22-hydroxy-docosanoic acid), triterpenes (lupeol and betulin), as well as carbohydrate derivatives (Table 4). Diacids and aromatics after Method 1 were detected in a smaller amount. This could be attributed to the fact that they are polymerized together and cannot been detected in GC/MS.

Suberin monomeric constituents were also determined by Method 2 (additional depolymerisation and silylation). Quantitative calculations were performed using standard calibration (as described in Section 3.5). The comparison of quantified compounds by Methods 1 and 2 is shown in Table 4.

After the Method 2 the main suberinic components were fatty acids (hexadecanoic acid ethyl ester, linoelaidic acid ethyl ester and ethyl stearate), hydroxyacids (20-hydroxyeicosanoic acid, 2-hydroxy-decenedioic acid and 22-hydroxy-docosanoic acid), diacids (dimethyl docosanedioate, 9,12-octadecadienoic acid and octadecanedioic acid), triterpenes (lupeol and betulin), as well as carbohydrate derivatives. It can be seen that by Method 2 more compounds can be detected than in Method 1 showing that after the first depolymerisation suberin has not been depolymerised completely or copolymerised during the drying operation. Especially, the increased amount of hydroxyacids and diacids after Method 2 showed that there were SA oligomers in the sample after the first depolymerisation and drying process.

According to the results obtained by Method 1 for the SA_U sample, the total yield of identified compounds was 53%, but after additional depolymerisation (Method 2), the yield increased to 91%. For the SA_T sample, a similar trend was observed, with 57% and 77% yields for Methods 1 and 2, respectively. These results point out that both suberin samples contain not only monomeric fractions but also oligomeric/polymeric fractions of compounds, which cannot be detected with GC-MS without an additional depolymerisation process before the sylilation step. As seen in Table 5 for the SA_T sample, the total yield of compounds per sample after Method 2 was lower than that for the SA_U sample. This can be explained by the suberin sample obtaining process where, for the SA_T sample an additional step in the workflow was included to precipitate polyphenols with FeCl_3_. As a result, some higher molecular fractions of SA and TPC were removed from the sample. This explains why the TPC content in SA was only 0.8% lower for SA_T, while the yield was 4.8% lower than that for SA_U samples (Table 1). To confirm that analysed SA samples also contain fractions of polymeric compounds, we performed GPC analysis, which is discussed in Section 2.3, Section 2.4 and Section 2.5.

### 2.3. Molar Mass Distribution in SA Samples

**Figure 4:**
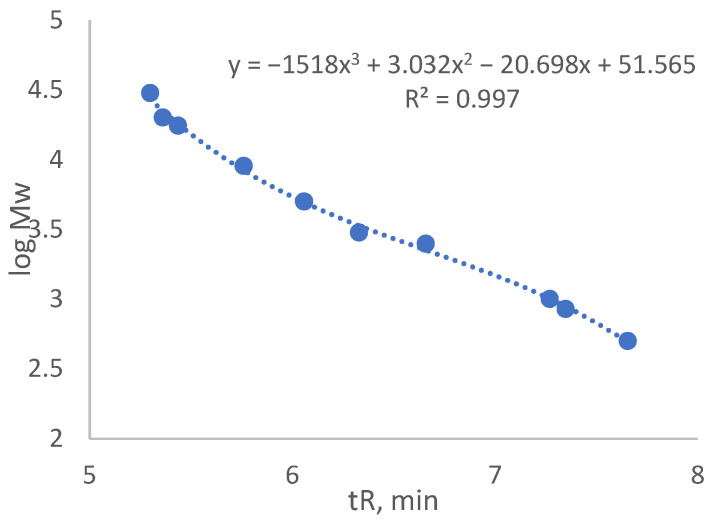
Polystyrene standard calibration graph after GPC-RID data.

The traditional method for calculating the molar mass of SA is sample fractionation with GPC and detection of the refractive index (RI). To see changes in molar mass distribution caused by SA treatment with FeCl_3_, GPC-RID analysis was performed for both SA_U and SA_T samples. Since no specific SA standard substances are commercially available (for each type of biomass from which the SA can be obtained, the composition of suberin and depolymerised SA can differ), the molar masses were assessed based on the polystyrene standard. The polystyrene calibration graph is shown in Figure 4.

**Figure 5:**
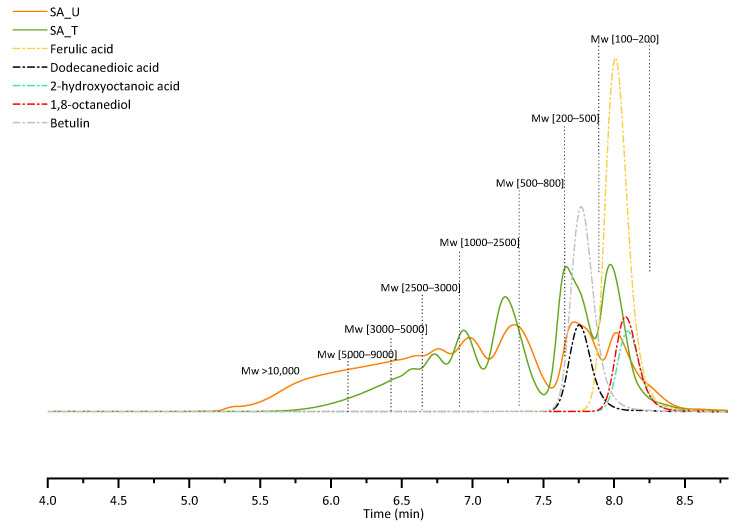
GPC-RID chromatograms of SA samples and standard substances.

GPC-RID chromatograms of both samples (treated and untreated) are presented in Figure 5. To show the retention times of the main compounds found in SA samples, several analyses of standard substances were also performed. Table 5 summarises the relative area percentage of each molar mass range determined with GPC-RID.

For both samples, the first two peaks in the chromatograms represent a low molecular weight fraction (Mw 100–500) of low dispersity, while the rest of the peaks correspond to higher molecular weights possessing a much wider distribution. For the untreated sample (SA_U), there is a noticeable shoulder on the low molecular weight peak, indicating that there are some components with Mw lower than 100 Da. This could indicate low molecular weight phenolic type compounds, and tannins, which are separated during the sample treatment, since this peak shoulder disappears after the treatment with FeCl_3_.

When comparing the distribution of different molecular weight fractions in all samples (Table 5), it can be seen that after the treatment (SA_T), a higher relative composition of low molecular weight fraction was observed, indicating that by removing tannins from sample, the high molecular weight fraction is either precipitated together with the tannin-FeCl_3_ complex, or prevented from forming by removing the tannins that can promote SA condensation.

Molar mass averages for the sample SA_U calculated from the calibration graph data were as follows: the number averaged molar mass (Mn) = 3601 ± 47 g·mol^−1^, and the weight averaged molar mass (Mw) = 7394 ± 150 g·mol^−1^. The calculated polydispersity of the analysed sample was 2.05.

Molar mass averages for the sample SA_T were Mn = 1707 ± 63 g·mol^−1^ and Mw = 2906 ± 117 g·mol^−1^. The calculated polydispersity of the analysed sample was 1.71.

Molar mass averages per sample showed the same tendencies as explained before—for SA_T sample, the number averaged molar mass and weight averaged molar mass were smaller than for the SA_U sample.

Even though GPC-RID and polystyrene calibration data were used, it was possible to calculate the molar mass for the SA samples. It is important to add that the quantitative aspect related to these results could be incorrect because of the major difference between the structures of suberin and polystyrene standards. That is why we also performed GPC analysis using MALS detector to calculate the absolute molar mass.

### 2.4. Fluorescence of the SA Samples

Absolute molar mass can be calculated using multi-angle light scattering (MALS). According to the definition of the absolute molar mass only the precise refractive index increment at chemical equilibrium (dn/dc) must be precisely known since there is a theoretically deduced relationship between the molar mass and the intensity of light scattered by macromolecules in a diluted solution. As a consequence, calibration standards are not necessary. The sample absorbed light at a high energy with short wavelength, and emitted light at a lower energy; thus, the molar mass could not be calculated. The MALS detector in LSIWC equipment (miniDAWN, Wyatt Technology) is a three-angle light scattering detector. To obtain more qualitative peaks the elimination of fluorescence is necessary; therefore, more lasers could be a solution because light filters can be used more effectively [23]. Therefore, for comparison, we sent SA samples to the Wyatt Technology Corporation in Germany (DAWN, Wyatt Technology) equipped with an 18-angle light scattering detector to be analysed there.

**Figure 6:**
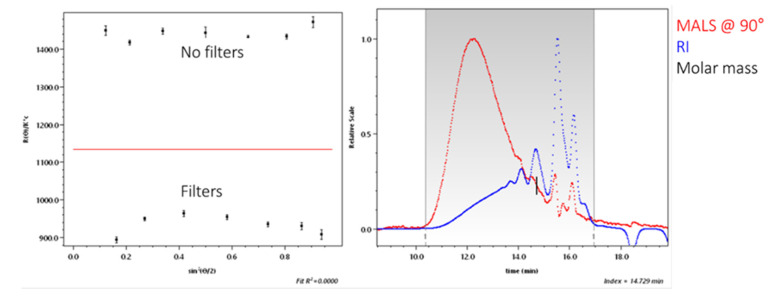
Intensity of the fluorescence for the sample SA_U.

At elution time ≈ 14.7 min there was a high concentration of sample and low molar mass, i.e., the area where fluorescence is relatively strong, but in this specific case the difference of molar mass was solely ≈300 g·mol^−1^. Grey box shows the analysed part of chromatogram (Figure 6). Only photodiodes with filters were used for data processing, so that the negligible effect of fluorescence could be removed.

**Figure 7:**
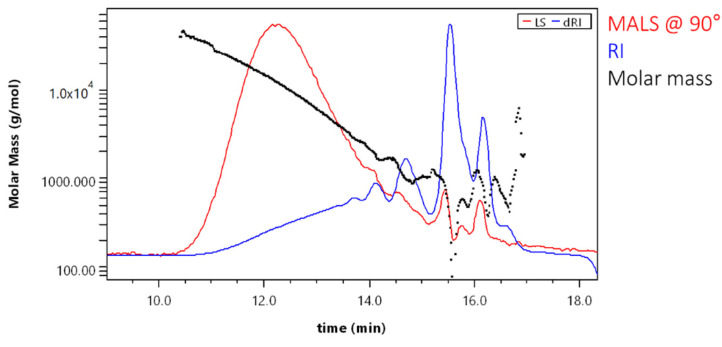
Molar mass versus elution volume plot for the sample SA_U.

Evenly decreasing molar mass allowed good GPC separation for a major part of the sample. Scattering of data points at the end of the chromatogram was due to low signal and overlapping peaks (Figure 7).

Molar mass averages for the sample SA_U when using the 18-angle light scattering detector were Mn = 650 ± 140 g·mol^−1^ and Mw = 2900 ± 100 g·mol^−1^. The polydispersity for the sample was 4.46. When comparing these results with those obtained with GPC-RID and polystyrene calibration (Mn = 3601 ± 47 g·mol^−1^ and Mw = 7394 ± 150 g·mol^−1^; polydispersity was 2.05), it can be seen that molar masses obtained by 18-angle MALD detector were lower (number averaged molar mass more than 5× and the weight averaged molar mass—more than 2× lower). Consequently, the polydispersity was lower. This indicates that using the RI detector for SA sample analysis resulted in higher calculated molar masses.

**Figure 8:**
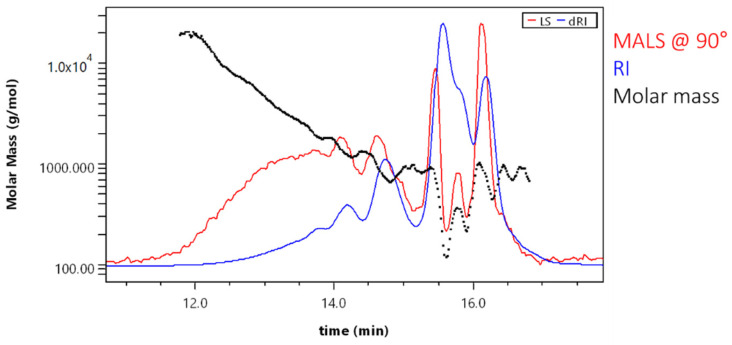
Molar mass versus elution volume plot (for sample SA_T).

An evenly decreasing molar mass proved good GPC separation for a major part of the sample (Figure 8).

Scattering of data points at the end of the chromatogram was due to low signal and overlapping peaks. Result fitting can partially eliminate this effect. Molar mass averages for the sample SA_T were Mn = 320 ± 30 g·mol^−1^ and Mw = 750 ± 40 g·mol^−1^. The polydispersity was 2.34. When comparing these results with those obtained with GPC-RID and polystyrene calibration (Mn = 1707 ± 63 g·mol^−1^ and Mw = 2906 ± 117 g·mol^−1^; polydispersity was 1.71), it can be seen that molar masses with 18-angle MALD detector were again lower (number averaged molar mass more than 5× and the weight averaged molar mass—more than 3× lower). Consequently, the polydispersity was also lower. This again indicates that the use the RI detector for SA sample analysis resulted in higher calculated molar masses.

**Figure 9:**
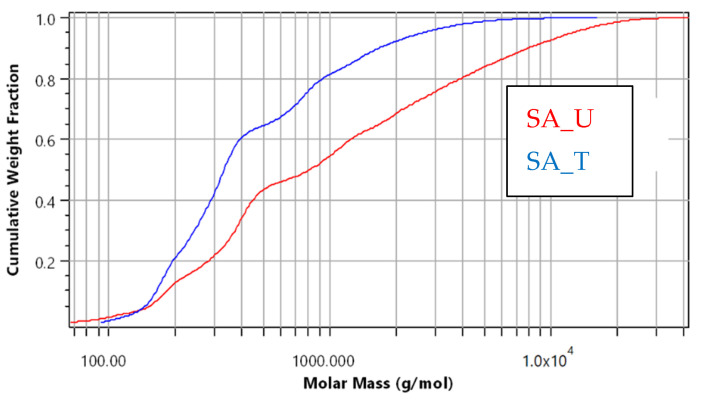
Molar mass distribution between SA_U and SA_T samples.

Cumulative molar mass distribution can be used for easy reading of fractions below or above a certain molar mass value (Figure 9). For example, the treated sample (SA_T) did not contain molecules with M > 10,000 g·mol^−1^, whereas before purification, the sample contained ≈ 10% molecules with M > 10,000 g·mol^−1^. This corresponds to the GPC-RID results (Table 5), indicating that SA_U samples had about 7% of fraction with M > 10,000 g·mol^−1^.

Infrared laser and fluorescence blocking filters eliminated the fluorescence of the analysed SA samples. Despite very low molar mass, a sufficient MALS signal was acquired, which allowed the determination of molar mass distribution. The GPC-MALS method revealed a shift of the molar mass distribution to lower values after sample purification. These results agree with GPC-RID data (same tendencies were be observed). The treatment of SA with FeCl_3_ led to samples with lower Mn values, while absolute values were lower when using GPC-RID and polystyrene calibration standards. This shows that for the successful analysis of these kinds of samples, a MALS detector equipped with 18 lasers and fluorescence filters is essential for successful absolute Mw determination. However, for screening tests GPC-RID results were sufficient to find optimal conditions.

### 2.5. MALDI-Tof Analysis of the SA Samples

**Figure 10:**
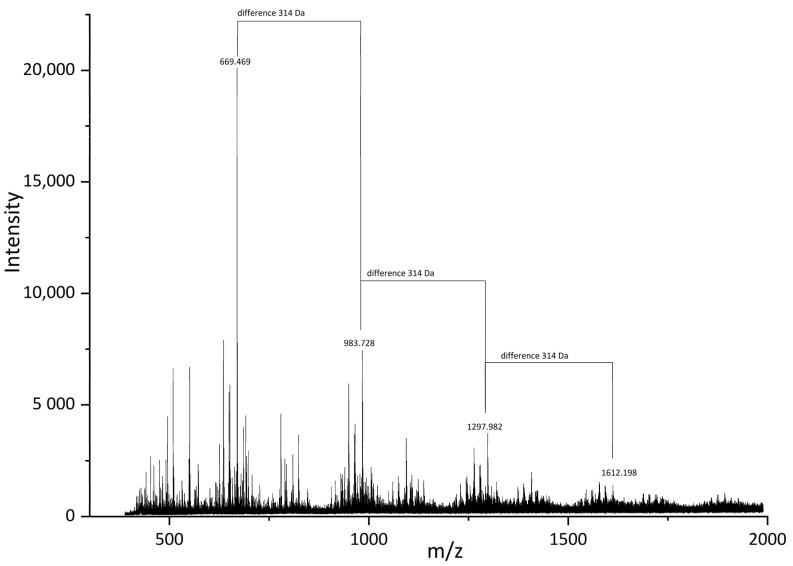
MALDI-Tof spectra of the SA_U sample.

For a better understanding of SA structures, we also performed MALDI-Tof analysis for SA_U sample (Figure 10). The main determined mases in the mass spectra were 669.47, 983.73, 1297.98 and 1612.20 Da. The difference between 983.73 and 669.47 Da was 314 Da, as well between 1612.20 and 1297.98 Da. The 314 Da mass corresponds to the monomeric unit of polymeric compounds in SA samples.

**Figure 11:**
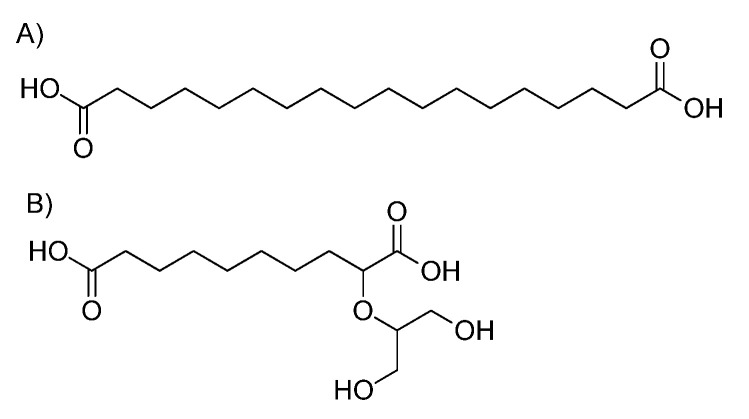
Possible monomeric compound structures (**A**) Octadecanedioic acid and (**B**) 2-(1,3-dihydroxyprop-2-oxy)decanedioic acid) in SA samples.

Octadecanedioic acid (Figure 11A) and 2-(1,3-dihydroxyprop-2-oxy)decanedioic acid (Figure 11B) are possible examples for a molecule with 314 Da mass. Of course, there are more possible structures for this monomeric unit. According to GC-MS data (Table 3 and Table 4), the monomeric unit should be either a hydroxyacid or diacid interlinked in the suberin structure with glycerol units. The structures found in SA samples are possibly three-dimensional and have a polymeric matrix.

## 3. Materials and Methods

### 3.1. Materials and Reagents

Materials and reagents were as follows: glacial acetic acid (AcOH), ≥99.8%; acetanhydride, ≥99%; diethyleneglycol (DEG), reagent grade, 98%; 4-(dimethylamino)pyridine (DMAP), reagent plus, ≥99%; N,N-dimethylformamide (DMF), ACS reagent, ≥99.8%, water content ≤150 ppm; hydrochloric acid (HCl), ACS reagent, ≥37%; nitric acid (HNO_3_), 69%; sulfuric acid (H_2_SO_4_), ACS reagent, 95.0–98.0%; acetone, ACS reagent, ≥99.5%; tetrahydrofuran (THF), anhydrous, ≥99.9%, inhibitor-free; methanol (MeOH), ACS reagent, ≥99.5%; ethyl acetate (EtOAc), anhydrous, 99.8%; dichloromethane (DCM), anhydrous, ≥99.8%; hexane, anhydrous, 95%; pyridine (Py), suitable for HPLC, ≥99.9%; dimethyl sulfoxide (DMSO), ACS reagent, ≥99.9%; 2-propanol (i-PrOH), ACS reagent, ≥99.5%; potassium hydroxide (KOH), ≥85%; sodium hydroxide (NaOH), ≥85%; iron(III) chloride, reagent grade, 97%; Silylating mixture III, for gas chromatography (GC) derivatization; 1.8-octanediol, ≥98%; myristic acid, Sigma Grade, ≥99%; 2-hydroxyoctanoic acid, ≥98%; dodecanedioic acid, ≥99%; betulin, ≥98%, and ferulic acid, ≥99%.

### 3.2. Suberinic Acid Isolation

**Figure 12:**
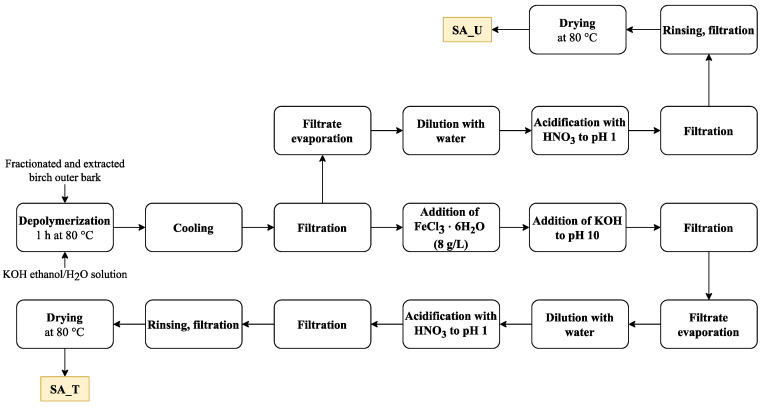
Schematic view of the SA isolation plan.

Isolated and fractionated birch outer bark was kindly supplied by AS Latvijas Finieris (Riga, Latvia). Birch outer bark samples were dried at room temperature (moisture content 4–5%) and milled in a SM 100 cutting mill (Retsch GmbH, Haan, Germany) to pass through a sieve with 4 mm aperture. Milled birch outer bark was fractionated by sieving using an AS 200 Basic vibratory sieve shaker (Retsch GmbH, Haan, Germany), and a fraction of 1–3.15 mm was collected. Fractionated birch outer bark was extracted with ethanol twice, as described by Godina et al. [4]. After extraction, birch outer bark with an o.d. substance content of about 45 % was used as a feedstock for depolymerisation (Figure 12).

About 2000 g (o.d. basis) extracted birch outer bark was depolymerised in potassium hydroxide (650 g) ethanol–water solution (20 L) for 1 h at 80 °C (extracted outer bark– ethanol–water–potassium hydroxide mass ratio 1:7.3:0.73:0.27). After depolymerisation, the solution was cooled down to 25 °C and filtered through 100 μm non-woven polyamide fabric. The depolymerisation filtrate was treated with FeCl_3_⋅6H_2_O (8 g per 1 L of filtrate) followed by addition of KOH to pH 10.0. The resulting precipitate was separated by filtration, and the resulting solution of depolymerised suberin was evaporated until 65 % of the solvent was recovered, followed by further dilution with 7 L of water. The obtained suspension was further acidified with HNO_3_ (69%) to pH 1.0 followed by filtration and rinsing with 11.5 L of deionised water and drying at 80 °C.

**Figure 13:**
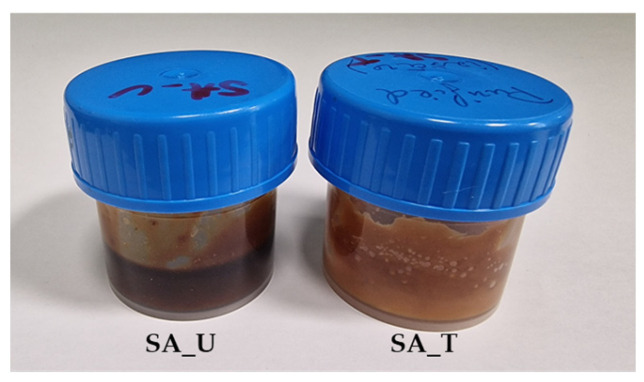
SA samples (SA_U and SA_T).

For comparison, an untreated SA sample was also obtained. In this case, the treatment with FeCl_3_⋅6H_2_O and KOH was skipped. The rest of the preparation steps were performed as described above. As a result, two SA samples were obtained: SA_T (treated with FeCl_3_⋅6H_2_O) (Yield 24.7 % o.d.m) and SA_U (untreated) (yield 29.3% o.d.m) (Figure 13).

### 3.3. SA Characterisation with Potentiometric Titration Methods

#### 3.3.1. Acid Number Determination

To about 0.2 g of the sample, 5 mL of DMSO was added and stirred for 1 h. Afterwards, 20 mL of i-PrOH and 5 mL of water were added, and the solution was titrated with 0.1 M KOH solution. Two replicate experiments were performed for each sample. The acid number was calculated according to Equation (1).

(1) $$\mathrm{Acid}\;\mathrm{number}=\frac{V_{\mathrm{analyte}}{\;\times \;C}_{\mathrm{KOH}\;}{\times \;M}_{\mathrm{KOH}}}{m}$$

V_analyte_—the volume of titrant (KOH) consumed for the analyte, mL; C_KOH_—the exact concentration of the titrant (KOH), mol·L^−1^; m–mass of the analysed sample, g; M_KOH_—KOH molar mass, g·mol^−1^.

#### 3.3.2. Epoxy Group Content Determination

To about 0.2 g of the sample, 10 mL of 0.2 M HCl in acetone was added. The solution was stirred for 1 h and titrated with a known concentration of 0.1 M KOH solution. The epoxy group content was calculated according to Equations (2) and (3).

(2) $$n_{\mathrm{epoxy},\frac{\mathrm{mmol}}{g}=\frac{{(V}_{\mathrm{Blank}}-V_{\mathrm{analyte}}{)\;\times \;C}_{\mathrm{KOH}}}{m}}$$

(3) $${\%}_{\mathrm{epoxy}}=\frac{n_{\mathrm{epoxy}}\times 15.9994}{10}$$

n_epoxy_—epoxy group amount per sample, mmol·g^−1^; V_Blank_—volume of titrant (KOH) consumed without analyte, mL; V_analyte_—the volume of titrant (KOH) consumed for the analyte, mL; C_KOH_—the exact concentration of the titrant (KOH), mol·L^−1^; m—mass of the analysed sample, g; 15.9994—molar mass of oxygen, g·mol^−1^;

### 3.4. Total Phenolic Content (TPC)

To about 1 g of the sample, 10 mL of DMSO was added. Glass tubes with the solutions were left at room temperature for 20 h and then placed in an ultrasonic bath for 60 min. To determine TPC, 7.9 mL of deionised water was added to plastic tubes, 0.1 mL of the SA solution, and 0.5 mL of Folin–Ciocalteu’s reagent was added. The solutions were conditioned in the dark for 8 min. Then, 1.5 mL of 20% Na_2_CO_3_ solution was added, and after 2 h of incubation in the dark, and the absorbance at 756 nm was measured. Gallic acid was used as a standard solution (0.05; 0.1; 0.3; 0.6; 0.8; 1.0 mg·mL^−1^) to determine the phenol content as gallic acid equivalents (GAE) mg·g^−1^ and further recalculated as total phenolic content (TPC, %) by mass (Equation (4)).

(4) $$\mathrm{TPC}=\frac{\frac{A-b}{a}}{10}\;\times \;\mathrm{DF}$$

A—absorption, au; a, b—coefficients of gallic acid calibration curve; DF—dilution factor.

### 3.5. FTIR Analysis

Data from a US Nicolet iS50 spectrometer (Thermo Fisher Scientific, Waltham, MA, USA) with a resolution of 0.2 cm^−1^, 32 scans were used to examine the SA structure. The attenuated total reflectance method using a diamond crystal prism was used to gather the FTIR data.

### 3.6. GC-MS Analysis of the SA Samples

After preparation according to methods 1 or 2, the sample (1 μL) was injected into a Thermo Scientific TRACE 1300 gas chromatograph with a Thermo Scientific ISQ quadrupole mass detector. A Thermo Scientific TG-5MS (30 m × 0.25 mm × 0.25 µm) column was used and an injector temperature of 250 °C, in spitless mode with a carrier gas (helium) flow of 1.20 mL·min^−1^. Oven temperature program: isothermally held at 150 °C for 5 min, then increased at 10 °C·min^−1^ and held for 1 min, before being finally increased at 2 °C·min^−1^ and passed at 300 °C for 15 min. The total time of analysis was 60 min. The transition line temperature of the mass detector was 250 °C, and the ion source temperature was 200 °C. The mass range was 45–700 Da. Each sample was analysed by two complementary methods:

Method 1: The SA sample was converted to the corresponding trimethylsilyl (TMS) derivatives and analysed quantitatively by GC-MS, allowing the identification of monomeric structures present in the mixture. The SA sample (approximately 50–100 mg) was reacted with 100 µL of Py, 200 µL silylating mixture III (1-(trimethylsilyl)imidazole/BSTFA/TMCS 3/3/2 (*v/v/v*)) for 20 min at 70 °C.

Method 2: To analyse the composition of the oligomeric/polymeric fraction of suberin, the sample before silylation was subjected to alkaline hydrolysis to release hydrolysable monomeric constituents. Approximately 50–100 mg of SA sample was dissolved in 1 mL of methanol, and then 1 mL of 1 M NaOH water solution was added. The sample was then incubated at 95 °C for 4 h. The mixture was cooled to room temperature, acidified to pH 3–3.5 with 1 M HCl, extracted three times with DCM, and the combined organic extracts were evaporated in a rotary evaporator. Finally, the obtained dry sample was trimethylsilylated as described above prior to GC-MS analysis.

To quantitatively identify the compounds in SA samples, we choose several standard substances covering different groups of chemical compounds:

(1) diols (1,8-octanediol)
(2) fatty acids and their corresponding esters (myristic acid)
(3) hydroxy acids and their corresponding esters (2-hydroxyoctanoic acid)
(4) diacids and their corresponding esters (dodecanedioic acid)
(5) extractives (betulin and lupeol)
(6) aromatics (ferulic acid)
(7) carbohydrates (D-glucouronic acid)
(8) other

The developed analytical method was partially validated in accordance with Eurachem validation guidelines [24] to ensure the method was fit for purpose. For Method 1 we determined linearity range, R^2^, LOD, and LOQ using the previously mentioned standards.

### 3.7. GPC-MALS-RID Analysis with Infrared Three-Angle Light Scattering Detector

The SA samples were analysed using Agilent 1260 Infinity HPLC system, equipped with isocratic pump, degasser and autosampler. For separation we used Plgel Mixed-E 300 × 8 mm column at 40 °C. An infrared three-angle light scattering detector miniDAWN^®^ and refractive index detector Optilab^®^ were used for detection. The thermostat temperature for the RI detector was 40 °C. The mobile phase was THF with a flow rate of 1.0 mL·min ^−1^. SA samples were prepared as THF solutions with 20 mg·mL^−1^ mass concentration and filtered through nylon syringe filters to remove undissolved solids (0.22 µm). The injection volume was 100 µL. The calculations for data obtained by GPC-RID were done using polystirol standards (with molar mass 500, 850, 1000, 2500, 3000, 5000, 9000, 17,500, 20,000 Da). All of the standard substances analysed with GC-MS were also analysed with GPC-MALS-RID.

### 3.8. GPC-MALS-RID Analysis with Infrared 18-Angle Light Scattering Detector

The SA samples were analysed using Agilent 1260 Infinity HPLC system, equipped with isocratic pump, degasser and autosampler. For separation 2 Plgel Mixed-E 300 × 8 mm columns were used at 40 °C. An infrared 18-angle light scattering detector DAWN^®^ with an infrared 785 nm laser and a refractive index detector Optilab^®^ with 785 nm light source were used for detection. The thermostat temperature for the RI detector was 40 °C. The mobile phase was THF with a flow rate of 1.0 mL/min. SA samples were prepared as THF solutions with 20 mg·mL^−1^ mass concentration and filtered through nylon syringe filters to remove undissolved solids (0.22 µm). The injection volume was 100 µL. The molar masses (Mn and Mw) for SA samples were calculated using dn/dc values.

### 3.9. MALDI-TOF Analysis

MALDI-TOF mass spectra were acquired with an UltrafleXtreme TOF–TOF mass spectrometer (Bruker Daltonics, Bremen, Germany) equipped with a 2000 Hz smartbeam-II laser (355 nm) using the positive ion reflectron mode. Panoramic pulsed ion extraction and external calibration were used for molecular weight assignment.

The dried droplet method was used in which the solutions of the sample (10 mg mL^−1^), matrix 2,5 dihydroxybenzoic (20 mg mL^−1^), and ionizing agent sodium trifluoroacetate (CF_3_COONa; 10 mg mL^−1^) in N,N-dimethylformamide were mixed in the volume ratio 4:20:1. The mixture (1 mL) was deposited on the ground-steel target.

## 4. Conclusions

The findings of this study show the importance of development of instrumental analytical methods for the comprehensive characterisation of suberin derived from plant biomass for the successful integration of suberinic products into biorefinery production chains.

SA can be removed from the birch outer bark upon alkaline depolymerisation, and produce a sample that is particularly enriched with diols, fatty acids and their esters, hydroxyacids and their esters, diacids and their esters, and extractives (mainly betulin and lupeol) and carbohydrates. The SA treatment with FeCl_3_ allows the possibility to obtain a sample that has a lower content of phenolic type compounds, while the obtained sample has lower molecular weight.

It was possible to identify the main monomeric units of SA samples by GC-MS system using direct silylation and additional depolymerisation step before silylation.

For molar mass distribution determination, it is important to perform GPC analysis. Even though chromatographic results can be obtained using a three-laser MALS detector, they are not fully correct because of the fluorescence of the SA samples. Therefore an 18-angle MALS detector with filters is more suitable for SA analysis.

MALDI-Tof analysis is a great tool for the polymeric compound identification, which cannot be done using GC-MS. From the MALDI data, we discovered that the main monomeric units that makes up the SA macromolecular structure are octadecanedioic acid and 2-(1,3-dihydroxyprop-2-oxy)decanedioic acid. This corresponds with the GC-MS results after depolymerisation indicating that hydroxyacids and diacids were the dominant type of compounds found in the sample.

## Figures and Tables

**Table 1:** SA sample chemical properties.

Sample	Acid Number, mmol·g^−1^	Epoxy Groups, mmol·g^−1^	TPC, %	Yield, %
SA_U	1.566 ± 0.005	0.59 ± 0.16	2.3 ± 0.4	29.3
SA_T	1.770 ± 0.005	0.66 ± 0.2	1.53 ± 0.10	24.7

**Table 2:** GC-MS method parameters.

Standard Group	Linearity Range, mg·mL^−1^	LOD, mg·mL^−1^	LOQ, mg·mL^−1^	R^2^	Calibration Equation
1	0.005–0.10	3.71·10^−4^	1.13·10^−3^	0.990	y = 9·10^9^x − 4·10^7^
2	0.005–0.10	7.84·10^−5^	2.38·10^−4^	0.97	y = 4·10^9^x − 3·10^7^
3	0.005–0.10	8.96·10^−5^	2.71·10^−4^	0.9990	y = 4·10^9^x − 6·10^6^
4	0.005–0.10	4.71·10^−5^	1.43·10^−4^	0.991	y = 5·10^9^x − 3·10^7^
5	0.02–1.00	9.54·10^−3^	2.89·10^−2^	0.997	y = 1·10^9^x − 5·10^7^
6	0.005–0.10	1.54·10^−3^	4.67·10^−3^	0.993	y = 3·10^8^x − 2·10^6^
7	0.005–0.10	4.47·10^−4^	1.36·10^−3^	0.94	y = 9·10^9^x − 6·10^6^

**Table 3:** Identification of monomers in SA samples detected by GC-MS (Method 1).

Peak	Compound	tR, min	Mw	Group
4	1,2-Cyclooctanediol	5.95	144.2	1
1	1-Octanecarboxylic acid	3.86	144.2	2
3	1-Heptanecarboxylic acid	5.27	140.2	2
6	Hexadecanoic acid	11.82	256.4	2
11	Icosanoic acid	15.74	312.5	2
16	Docosanoic acid	18.37	340.6	2
2	Nonanoic acid ethyl ester	4.25	186.3	2
14	Hexadecanoic acid ethyl ester	16.87	284.5	2
15	Linoelaidic acid ethyl ester	17.32	310.4	2
21	Ethyl stearate	21.8	312.5	2
20	20-Hydroxyeicosanoic acid	20.82	328.5	3
22	2-Hydroxy-decanedioic acid	22.59	218.3	3
23	22-Hydroxy-docosanoic acid	24.81	356.6	3
24	Dimethyl docosanedioate	25.02	258.3	4
5	Nonanedioic acid	8.96	188.2	4
8	Pentanedioic acid	13.47	132.1	4
9	Hexanedioic acid	14.37	146.1	4
10	Dodecanedioic acid	15.25	230.3	4
12	Hexadecanedioic acid	16.27	286.4	4
13	9,12-Octadecadienoic acid	16.72	280.4	4
17	Octadecanedioic acid	19.02	314.5	4
18	11,14-Eicosadienoic acid	20.29	308.5	4
19	1,8-Octanedicarboxylic acid	20.38	202.3	4
25	Lupeol	34.16	426.7	5
26	Betulin	38.39	442.7	5
7	3-(3-Hydroxy-4-methoxyphenyl)acrylic acid	12.48	194.2	6
	Carbohydrate derivatives			7

**Table 4:** Quantification of monomers in SA samples detected by GC-MS by Method 1 and 2.

Peak	Compound		Group	Amount, %			
		Mw		Met. 1	Met. 2	Met.1	Met. 2
				SA_T		SA_U	
4	1.2-Cyclooctanediol	144.2	1	0.07	0.04	0.06	0.03
Total				0.07	0.04	0.06	0.03
1	1-Octanecarboxylic acid	144.2	2	0.20	0.64	0.29	0.88
3	1-Heptanecarboxylic acid	140.2	2	0.21	0.67	0.31	0.92
6	Hexadecanoic acid	256.4	2	0.21	0.68	0.31	0.93
11	Icosanoic acid	312.5	2	0.12	0.37	0.17	0.50
16	Docosanoic acid	340.6	2	0.23	0.75	0.34	1.02
2	Nonanoic acid ethyl ester	186.3	2	0.05	0.15	0.07	0.20
14	Hexadecanoic acid ethyl ester	284.5	2	0.34	1.07	0.49	1.48
15	Linoelaidic acid ethyl ester	310.4	2	0.93	2.97	1.36	4.05
21	Ethyl stearate	312.5	2	2.66	4.81	2.60	5.98
Total				4.95	12.11	5.94	15.96
20	20-Hydroxyeicosanoic acid	328.5	3	0.89	1.61	0.87	2.00
22	2-Hydroxy-decanedioic acid	218.3	3	5.04	9.10	4.93	11.32
23	22-Hydroxy-docosanoic acid	356.6	3	2.59	4.68	2.54	5.82
Total				8.52	15.40	8.34	19.14
24	Dimethyl docosanedioate	258.3	4	0.84	1.25	0.75	3.79
5	Nonanedioic acid	188.2	4	0.06	0.08	0.05	0.25
8	Pentanedioic acid	132.1	4	0.14	0.21	0.12	0.63
9	Hexanedioic acid	146.1	4	0.35	0.52	0.31	1.58
10	Dodecanedioic acid	230.3	4	0.14	0.20	0.12	0.61
12	Hexadecanedioic acid	286.4	4	0.21	0.31	0.19	0.95
13	9,12-Octadecadienoic acid	280.4	4	0.87	1.30	0.77	3.92
17	Octadecanedioic acid	314.5	4	0.37	0.55	0.33	1.65
18	11,14-Eicosadienoic acid	308.5	4	0.35	0.52	0.31	1.58
19	1,8-Octanedicarboxylic acid	202.3	4	0.17	0.25	0.15	0.77
Total				3.49	5.20	3.10	15.74
25	Lupeol	426.7	5	7.15	7.84	6.48	7.10
26	Betulin	442.7	5	25.80	28.28	23.38	25.64
Total				32.95	36.12	29.86	32.74
7	3-(3-Hydroxy-4-methoxyphenyl)acrylic acid	194.2	6	0.73	0.98	0.45	0.74
Total				0.73	0.98	0.45	0.74
	Carbohydrate derivatives		7	6.23	7.57	5.74	6.87
Total				6.23	7.57	5.74	6.87
Total per sample, %				56.94	77.42	53.49	91.22

**Table 5:** GPC-RID (molar mass range and relative area percentage) data for SA samples.

Sample	Molar Mass Range, Da						
	100–200	200–500	500–800	1000–2500	2500–3000	3000–5000	>10,000
	Relative Area Percentage, %						
SA_U	19	14	14	10	17	19	7
SA_T	24	23	22	10	16	5	-
